# Human-viral chimera: a novel protein affecting viral virulence and driving host T-cell immunity

**DOI:** 10.1038/s41392-020-00272-x

**Published:** 2020-08-25

**Authors:** Zhenling Wang, Li Zhang, Min Wu

**Affiliations:** 1 State Key Laboratory of Biotherapy and Cancer Center, West China Hospital, Sichuan University, and Collaborative Innovation Center of Biotherapy, 610041 Chengdu, China; 2 Department of Biomedical Sciences, University of North Dakota School of Medicine and Health Sciences, Grand Forks, ND USA

**Keywords:** RNA splicing, Infection

**Fig. 1:**
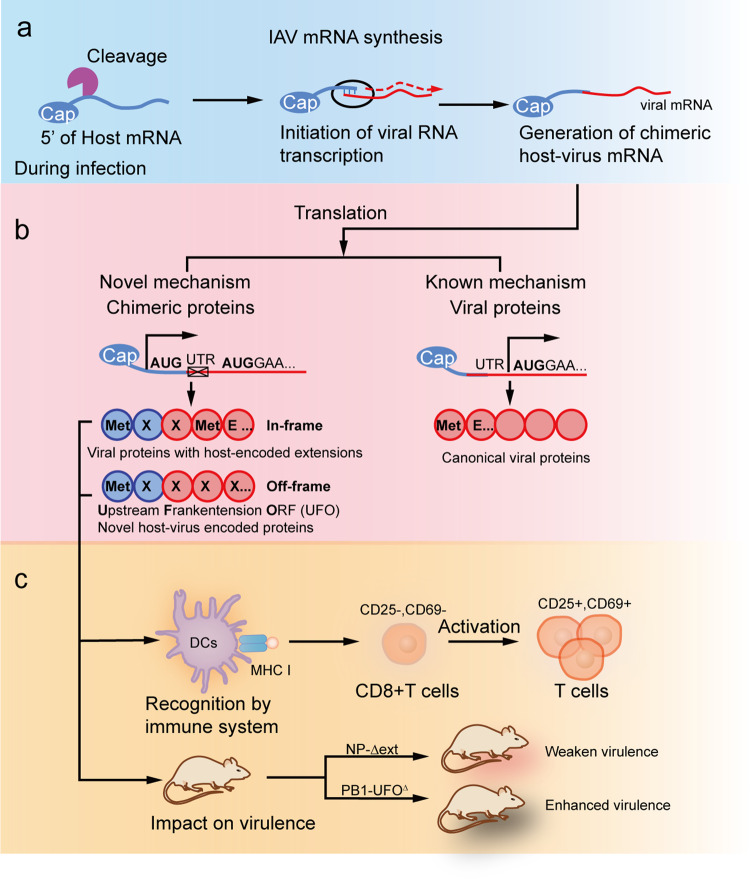
The chimeric proteins produced by the novel mechanism activate T-cell responses and affect virulence. **a** The synthesis of influenza A virus (IAV) mRNA; **b** The formation of host-viral chimeric proteins in the presence of upstream AUGs (uAUGs); **c** Host-virus protein chimeras are recognized by T cells and affect virulence in mice.

Recently, a paper published in *Cell* by Ho et al. describes an interesting finding how a virus can highjack the host machinery and gene sequence to synthesize its own virulence proteins to facilitate invasion, replication, and spread. The research reveals that RNA viruses like influenza A virus (IAV) can produce previously unrecognized chimeric proteins containing both viral and human genetic information, which can then affect virulence and modulate T cell responses in hosts (Fig. 1).^1^

Host mRNA plays an indispensable role in the life cycle of several highly pathogenic RNA viruses. The segmented negative-strand RNA viruses (sNSVs) can make direct use of the 5′ termini of host mRNAs during transcribing their own genes. In sNSVs, the synthesis of viral mRNA is initiated by using short 5′-methyl-7-guanosine (m7G) capped RNA sequences, which are cleaved by the viral polymerase from the host RNA polymerase II (RNAPII) transcript. This process is called “cap-snatching”.^2^ Cap-snatching initiates virus gene transcription to form genetic hybrids of host and virus mRNAs. In the latest *Cell* issue (25 June 2020), Ho et al.^1^ report that IAV, which belongs to sNSVs, can obtain functional upstream start codons (uAUGs) by appropriating 5′ terminal mRNA sequences from their hosts, and this mechanism is termed “start-snatching”, reflecting the feature of grabbing start codon-associated sequences. In chimeric host-viral transcripts, translation from host-derived uAUGs would access upstream viral ORFs (uvORFs).

Ho et al. determined the abundance of uAUGs in cap-snatched host sequences archived in a DEFEND-seq dataset that had previously been generated from A549 cells infected with IAV.^2^ These authors observed that the distributions of cap-snatched sequences containing uAUGs, which were present in each IAV genome segment are similar to all cap-snatched sequences.^1^ The team hence proposed for the first time that viruses can form chimeric RNAs with hybrid coding potential upon infection.

To confirm whether the IAV sequences within the untranslated regions (5′ UTRs) lacked stop codons, Ho et al. first used the NCBI Influenza Virus database to perform bioinformatic analysis on all IAV H1N1 strains and determined the nucleotide sequence variability of the 5′ UTRs.^3^ Their results suggest that 5′ UTRs of each individual IAV genome segment are highly conserved and can maintain a reading frame in at least one frame.^1^ The upstream stop codons were absent from IAV sequences within the 5′ UTRs of genome segments when these were in-frame (PB2, HA, NP, NA, and NS) and out-of-frame (PB2, PB1, PA, NA, M, and HA) with the major ORF.^1^ Their analyses strongly indicate that in the presence of uAUGs, these segments can code for N-terminally extended viral proteins or make novel hybrid polypeptides. To test this hypothesis, the authors translated viral sequences that had cap-snatched uAUGs, and found that uvORFs were present in all genome segments, and generated polypeptides of varying lengths when licensed by uAUG-containing RNAs.^1^ To identify whether uAUGs initiate translation of viral 5′ UTRs, Ho et al.^1^ performed ribosomal profiling of IAV infected cells and found that ribosome-protected fragments (RPFs) were mapped to both the human and viral genomes. At the same time, they have observed that ribosomes were accumulated at the canonical initiation site, supporting that translation is initiated in this region.

To biochemically identify the chimeric proteins existing during IAV infection, Ho et al.^1^ performed mass spectrometry analyses of cell lysates and characterized three proteins PB1-UFO, PB2-UFO, and NP-ext, which are present at a moderate abundance within infected cells.^1^ Furthermore, they detected NP-ext in purified virions, but it is unclear whether influenza virions can specifically package other uvORF-encoded proteins. The authors next studied whether the host’s immune system can recognize chimeric host-viral proteins. IAVs were modified by inserting OVAI (SIINFEKL), which is a class I-restricted peptide epitope of ovalbumin (OVA) and can be used to detect a strong CD8+ T cell response.^4^ The sequences encoding OVAI were directly integrated into the UTR of PB1 segment (frame 3 uvORF) to encode PB1-UFO (SIIN). At the same time, to determine whether uvORFs are translated by default without being interrupted by stop codons, the authors deleted the stop codons of NS segment (NS, frame 2 uvORF) and then inserted OVAI into the extended uvORF to encode NS-UFO (SIIN). CD8+ T cells from the transgenic OT-I mice were activated when incubated with DC2.4 cells infected with PB1-UFO (SIIN) virus and bone-marrow-derived dendritic cells (BMDCs) infected with the NS-UFO(SIIN) virus (Fig. 1), suggesting that in the absence of stop codons, uvORFs are expressed and recognized by T cells during infection. Subsequently, to probe the impact of the chimeric host-viral proteins on viral pathogenesis, they constructed recombinant viruses and selected NP-Δext and PB1-UFO^Δ^ to infect BALB/c mice. The NP-Δext viruses were less virulent while the PB1-UFO^Δ^ viruses displayed increased virulence (Fig. 1). Together, these results elegantly demonstrate that during IAV infection, the adaptive immune system can detect the expression of uvORFs, which can modulate the pathogenic severity.

To extend this finding to a universal mechanism for viral pathogenesis, the authors tested whether NP-ext and PB1-UFO are conserved across different strains.^1^ By analyzing sequences of the IAV subtypes (H1N1, H3N2, and H5N1), they found that PB1-UFO is conserved within each of these three virus subtypes.^1^ Finally, Ho et al. attempted to confirm that start-snatching-mediated novel ORFs could be generalized from IAV to many other sNSVs. By employing the member of the *Orthomyxoviridae* family, influenza B virus (IBV) and other families of sNSV, Lassa virus (LASV),^5^ the authors performed sequence analysis and showed that the chimeric host-virus proteins could be ubiquitous from IAV to other sNSVs.

In summary, this study reveals the universal existence of a novel mechanism employed by IAV to generate chimeric host-virus mRNAs with coding potential during infection. Moreover, this mechanism appears to be a common virulence strategy for other family members of sNSVs. This finding of start-snatching phenomenon is important for scientific communities to further understand the molecular pathogenesis of RNA viruses and probably other pathogens in the infected host, lending critical insight into designing novel approaches to control emerging viral infections, such as SARS-CoV-2.
